# A deterministic map of Waddington's epigenetic landscape for cell fate specification

**DOI:** 10.1186/1752-0509-5-85

**Published:** 2011-05-27

**Authors:** Sudin Bhattacharya, Qiang Zhang, Melvin E Andersen

**Affiliations:** 1 Division of Computational Biology, Program in Chemical Safety Sciences, The Hamner Institutes for Health Sciences, Research Triangle Park, NC 27709, USA

## Abstract

**Background:**

The image of the "epigenetic landscape", with a series of branching valleys and ridges depicting stable cellular states and the barriers between those states, has been a popular visual metaphor for cell lineage specification - especially in light of the recent discovery that terminally differentiated adult cells can be reprogrammed into pluripotent stem cells or into alternative cell lineages. However the question of whether the epigenetic landscape can be mapped out quantitatively to provide a predictive model of cellular differentiation remains largely unanswered.

**Results:**

Here we derive a simple deterministic path-integral quasi-potential, based on the kinetic parameters of a gene network regulating cell fate, and show that this quantity is minimized along a temporal trajectory in the state space of the gene network, thus providing a marker of directionality for cell differentiation processes. We then use the derived quasi-potential as a measure of "elevation" to quantitatively map the epigenetic landscape, on which trajectories flow "downhill" from any location. Stochastic simulations confirm that the elevation of this computed landscape correlates to the likelihood of occurrence of particular cell fates, with well-populated low-lying "valleys" representing stable cellular states and higher "ridges" acting as barriers to transitions between the stable states.

**Conclusions:**

This quantitative map of the epigenetic landscape underlying cell fate choice provides mechanistic insights into the "forces" that direct cellular differentiation in the context of physiological development, as well as during artificially induced cell lineage reprogramming. Our generalized approach to mapping the landscape is applicable to non-gradient gene regulatory systems for which an analytical potential function cannot be derived, and also to high-dimensional gene networks. Rigorous quantification of the gene regulatory circuits that govern cell lineage choice and subsequent mapping of the epigenetic landscape can potentially help identify optimal routes of cell fate reprogramming.

## Background

**Figure 1:**
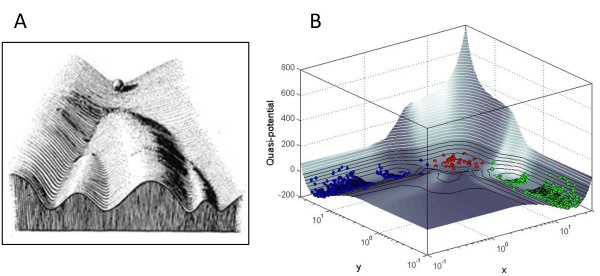
**Mapping Waddington's epigenetic landscape**. **(A) **The "epigenetic landscape" proposed by Conrad Waddington shows a ball rolling down valleys separated by ridges on an inclined surface, as a visual metaphor for the branching pathways of cell fate determination. Figure reproduced from original text by Waddington [5]. **(B) **The computed epigenetic landscape for a two-gene (*x *and *y*) regulatory network with mutual inhibition and positive autoregulation, where the elevation represents a path-integral quasi-potential derived from the deterministic rate equations describing the interactions of the two genes. We show that the "valleys" on this computed surface correspond to stable steady states (attractors) of the network, while the "ridges" separating the valleys represent barriers to stochastic transitions among multiple steady states. Colored circles represent a population of stochastically simulated "cells" (multiple instances of the network) residing in different stable steady states.

The biologist Conrad Hal Waddington, in the course of a career spanning four decades (1930s - 1970s), attempted a bold synthesis of the fields of genetics, embryology and evolution [1,2]. The centerpiece of his vision was the idea of the "epigenetic landscape", first described in *An Introduction to Modern Genetics *[3], and elaborated in subsequent monographs [4,5]. Waddington portrayed the epigenetic landscape as an inclined surface with a cascade of branching ridges and valleys (Figure 1A), which in the context of cell lineage selection, represent the series of "either/or" fate choices made by a developing cell. He envisioned that on this landscape, "the presence or absence of particular genes acts by determining which path shall be followed from a certain point of divergence [1,4]", thus providing in a single image an appealing, and influential, metaphor for the connection between genotype and phenotype.

In the quantitative view of a cell as a dynamical system governed by genetic interaction networks [6], an intuitive association can be made between the valleys ("creodes" in Waddington's terminology) on the epigenetic landscape and the trajectories leading to the *attractors*, or stable steady states, of the gene networks that regulate cell fate [7-9]. But can we quantitatively map the undulating surface of the landscape, thereby providing a predictive model of the "directionality" of cellular differentiation? Waddington himself cautioned that the epigenetic landscape, while useful as a "rough and ready picture" of development, "cannot be interpreted rigorously [5]". The mathematician René Thom, in his formulation of *catastrophe theory *inspired by Waddington's ideas, proposed that a generalized "potential surface" could be derived for any dynamical system [2,10]. However Thom's later writings suggest that he did not believe it possible to quantify the epigenetic landscape [11]. This view has been echoed by other authors, who have described the landscape as a "colorful metaphor [2]" with "no grounding in physical reality [1]".

Huang, Wang and colleagues have recently proposed a probabilistic "pseudo-potential" to quantify the epigenetic landscape for a gene network regulating cell fate, where the elevation of the surface is inversely related to the likelihood of occurrence of a particular state in phase space [8,12,13]. In this formulation a stochastic potential energy landscape is characterized for a gene network, based on a Hartree mean-field approximation of the underlying master equation [14]. Such stochastic formulations have also been used to derive probabilistic potential landscapes for the lysis-lysogeny switch in bacteriophage lambda [15-17], the mitogen-activated protein kinase (MAPK) signal transduction network [18], biochemical oscillations [19], and the predator-prey system [20].

Here we propose a simple numerical method to map the epigenetic landscape that is not based on a probabilistic or master-equation approach. Instead, a quasi-potential surface (Figure 1B) is derived directly from the deterministic rate equations governing the dynamic behavior of a gene regulatory circuit. We then use stochastic simulations to show that the elevation of this computed landscape correlates to the likelihood of occurrence of particular cell fates, with well-populated low-lying valleys representing stable cellular states and higher ridges acting as barriers to transitions between the stable states.

Finally, we discuss ways in which this quantitatively mapped landscape may help predict the efficiency of cellular de-differentiation or trans-differentiation, and identify optimal routes of cell fate reprogramming. Recent discoveries have challenged the dogma of cell fate determination as a unidirectional and irreversible process. Even terminally differentiated adult cells have now been shown to retain considerable phenotypic plasticity and the ability to be reprogrammed into pluripotent stem cell-like states [21-27] or into alternative differentiated lineages [28-34] by forced expression of a single gene or a small number of genes. These findings have led to a resurgence of interest in Waddington's ideas about cell lineage choice, with several authors invoking the image of the epigenetic landscape [7-9,35-39]. However the theoretical basis of plasticity in cell fate is still not fully understood, and the efficiency of reprogramming in these studies is often quite low [36]. A quantitative understanding of the "forces" that drive cell differentiation, and the "barriers" that separate stable cell states, is urgently needed. Such understanding may eventually enable us to predict the relative ease or difficulty of de-differentiation or trans-differentiation among multiple cellular states.

## Results and Discussion

### Derivation of the quasi-potential landscape

We first illustrate our quantitative approach with a simple circuit of two genes *x *and *y *that inhibit each other, forming a double-negative feedback loop structure (see **Methods**). This circuit works as a toggle switch with two stable steady states: one state with high *y *and low *x *expression, and the other state with high *x *and low *y *expression [40]. Such "bistable" switches formed by mutual antagonism of a pair of key regulatory genes underlie many binary cell fate choices [7,13]. The circuit can be described as a two-variable dynamical system, with the rate of change in expression of each of the two genes given as a function of their expression levels:(1)

If we were able to derive a closed-form potential function *V(x,y) *for the system in Eq. 1 that satisfied the conditions:(2)

then the local minima on the two-variable potential surface *V(x,y) *would correspond mathematically to the stable steady states of the system, given that at the local minima on the surface (∂*V*/∂*x *= 0; ∂*V*/∂*y *= 0), the rates of change in expression of both genes *x *and *y *would be zero (per Eq. 2). But such a closed-form potential function can be derived only in the case of a *gradient system*, defined by the condition [41]:(3)

In general, condition (3) will not be valid for an arbitrary circuit of two genes *x *and *y *that regulate each other as per Eq. 1, making it impossible to derive a closed-form potential function.

**Figure 2:**
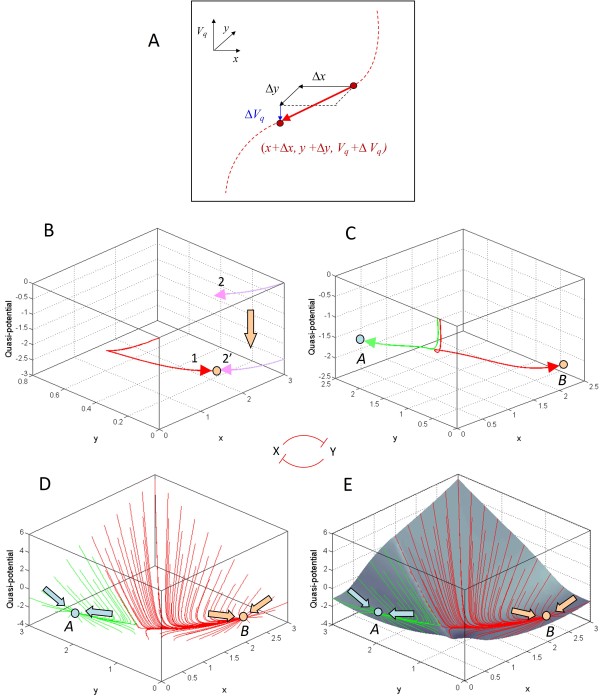
**Computing the epigenetic landscape for a bistable switch based on a double-negative feedback circuit of two genes *x *and *y***. **(A) **Paths followed by a simulated cell on the epigenetic landscape are obtained by integrating the change in quasi-potential Δ*V*_*q *_(Eq. 4 in text) along a trajectory as a function of time. (*x*+Δ*x*) and (*y*+Δ*y*) give the new position at each step along the trajectory in *x-y *phase space, while (*V*_*q*_+Δ*V*_*q*_) gives the new elevation on the quasi-potential surface. The initial value of the quasi-potential at the start of any individual trajectory is arbitrarily set to zero. **(B) **Two trajectories (*1 *and *2*) that converge to the same attractor on the *x-y *phase plane are aligned vertically so that both trajectories also converge to the same quasi-potential level. **(C) **Two trajectories that originate at adjacent points on the phase plane but converge to different attractors *A *and *B *are aligned vertically so that the initial quasi-potential levels of the two trajectories are equal. **(D) **Multiple trajectories starting from different points on the *x-y *phase plane are then aligned as described in panels **B **and **C**. To identify distinct basins of attraction, trajectories are shown colored according to the attractor to which they converge (arrows). This two-gene double-negative feedback circuit produces a bistable system with two attractors *A *and *B*. **(E) **Finally, interpolation among multiple trajectories aligned across the phase plane produces the epigenetic landscape.

Therefore, given that a gene circuit is in general a *non-gradient system*, we define a term *V*_*q *_that changes incrementally along a trajectory followed by the system in *x-y *phase space (Figure 2A) as follows:(4)

where Δ*x *and Δy are sufficiently small increments along the trajectory such that  and  can be assumed to remain unchanged over the interval [(*x*, *x*+Δ*x*); (*y*, *y*+Δ*y*)]. The quantities Δ*x *and Δy are obtained as the products  and , respectively, where Δ*t *is the time increment. We use the term "quasi-potential" to describe *V*_*q*_, to emphasize its distinction from a closed-form potential function.

The change in the quasi-potential, Δ *V*_*q*_, can be rewritten from Eq. 4 as:(5)

For positive increments in time Δ*t*, Δ *V*_*q *_is thus always negative along an evolving trajectory, ensuring that trajectories flow "downhill" along a putative "quasi-potential surface". Stable steady states of the system (*dx*/*dt *= 0; *dy*/*dt *= 0) would correspond to local minima on this quasi-potential surface, given that at these states Δ *V*_*q *_= 0 (per Eq. 5). The overall change in the quasi-potential along a trajectory can then be calculated by numerically integrating the quantity Δ *V*_*q *_in Eq. 4 from a given initial configuration up to a stable steady state, thereby allowing us to map out a temporal trajectory along the putative quasi-potential surface (Figure 2B). The quasi-potential thus defined is a measurable quantity that is minimized along a trajectory from any initial condition to an attractor in the phase space of the two genes, and is in effect a Liapunov function of the dynamical system represented by the two-gene circuit [41].

**Figure 3:**
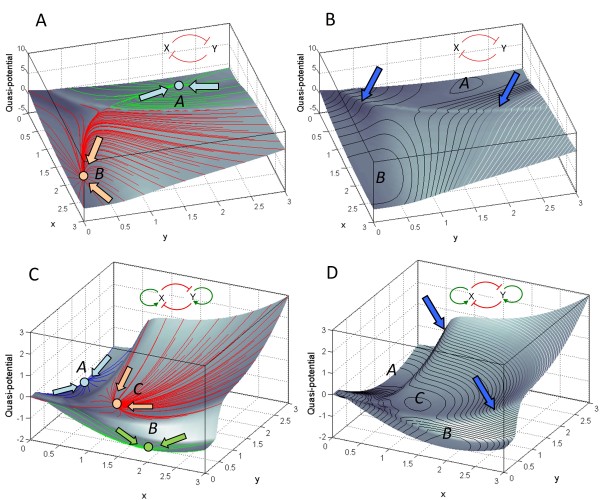
**Ridges and valleys on the computed epigenetic landscape of a bistable (A, B) and a tristable (C, D) regulatory network of two genes *x *and *y***. The alignment of trajectories produces the "ridges" on the epigenetic landscape (indicated by arrows in panels **B **and **D**) that separate the "valleys", or basins of attraction of multiple stable states of the network (points *A*, *B *and *C*). Equi-potential lines are drawn on the landscape to depict the curvature of the surface. In addition to the double-negative feedback loop between genes *x *and *y *that produces the bistable network (panels **A **and **B**), the tristable network (panels **C **and **D**) requires additional positive autoregulation of the two genes [8,13,42] (see **Methods**).

The procedure described above was repeated to evaluate the change in the quasi-potential along trajectories originating from different points in *x-y *phase space. To derive a quasi-potential surface from multiple trajectories, we then make the following assumptions: (i) two trajectories with different initial conditions that converge to the same steady state must also converge to the same final quasi-potential level (Figure 2B); (ii) two trajectories that originate from "adjacent" initial conditions that are sufficiently close in *x-y *phase space, but converge to different steady states, must start from the same initial quasi-potential level (Figure 2C). Observation (i) allows us to map out a basin of attraction from multiple trajectories converging to a single steady state; while observation (ii) enables the alignment of two adjacent basins of attraction along their shared basin boundary, or *separatrix*. (Essentially, (i) and (ii) together amount to the assumption that the putative epigenetic landscape is continuous.) The quasi-potential surface can then be obtained by interpolation among the aligned trajectories (Figure 2D, E), yielding the epigenetic landscape with its characteristic ridges and valleys (Figure 3A, B).

The same procedure can be applied to systems with more than two stable steady states - for instance, a "tristable" system produced by a circuit of two genes that induce their own expression, in addition to mutual inhibition (Figure 3C, D). This system has three steady states - two of which represent alternative differentiated cell lineages, while the third state depicts the common progenitor cell of the two lineages [8,13,42].

### Quantitative interpretation of the quasi-potential landscape

**Figure 4:**
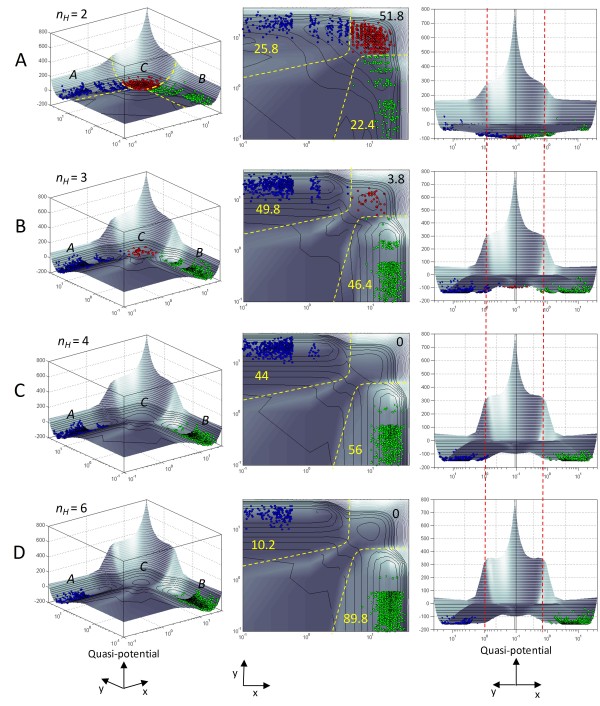
**Valleys on the computed epigenetic landscape represent high-occupancy stable steady states, while ridges represent barriers to stochastic transitions between those stable states**. For the tristable two-gene system, increasing the Hill coefficient *n*_*H*_, which represents the degree of ultrasensitivity in autoregulation and mutual inhibition of the two genes (see **Methods**), makes the ridges (barriers) higher and steeper relative to the valleys (attractors). Higher ridges reduce the probability of stochastic switching among adjacent attractors. **(A) ***n*_*H *_= 2; (**B) ***n*_*H *_= 3; **(C) ***n*_*H *_= 4; **(D) ***n*_*H *_= 10. **Left Panels**: Colored circles represent a population of 1000 stochastically simulated "cells" residing in the three stable steady states *A *(blue), *B *(green) and *C *(red). States *A *and *B *represent two alternative differentiated cell fates, and state *C *their common progenitor state [8,13,42]. All simulations were started from state *B *as the initial condition, and run to time *t *= 10,000 (dimensionless units). As the ridges separating the steady states grow higher, fewer cells are able to escape state B for states A and C through stochastic fluctuations. **Middle Panels**: Projections of the epigenetic landscape onto the *x-y *phase plane. Numbers refer to the percentage of simulated cells residing in the respective steady states. Dashed yellow lines show boundaries between the basins of attraction of the steady states. **Right Panels**: An alternative view of the epigenetic landscape. The vertical dashed red lines are guides to the eye to show that the relative distance between the steady states on the x-y phase plane does not change appreciably even as the Hill coefficient *n*_*H *_is increased from 2 to 6. The change in relative occupancy of the attractors can therefore be attributed to the increased height and steepness of the barriers separating them.

**Figure 5:**
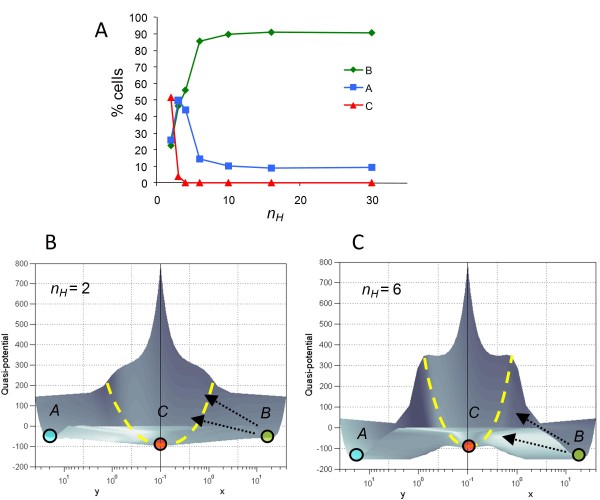
**Height and steepness of barriers affects stochastic occupancy of stable states**. **(A) **Percentage of stochastically simulated cells in the three attractors *A, B *and *C *in the tristable two-gene system at time *t *= 10,000 for a range of values of the Hill coefficient *n*_*H*_. All simulations were started from state *B *as the initial condition, and run to time *t *= 10,000 (dimensionless units). **(B, C) **With increasing *n*_*H*_, the height and steepness of the ridges relative to the valleys is increased, making stochastic transitions (arrows) from state *B *to state *C*, and thereafter to state *A*, less likely. (At longer time scales, where the distribution of the cell population among various attractors approaches an equilibrium, the percentage of cells in each attractor correlates simply to the relative depth of the attractor: see Figure S1, Additional File 1.) **(B) ***n*_*H *_= 2; **(C) ***n*_*H *_= 6.

To establish that the "elevation" of the computed landscape at a given location in *x-y *phase space correlates inversely to the probability of occurrence of the corresponding network state, we used stochastic simulations [43] of the underlying gene network. These simulations, which take into account fluctuations in gene expression levels [44] in a population of simulated "cells" (multiple instances of the gene network), showed that the "valleys" of low elevation on the computed epigenetic landscape correspond to stable cellular states, with "deeper" valleys associated with higher probability of occupancy than shallower valleys (Figure 4A-D). On the other hand, the "ridges" separating the valleys represent barriers to stochastic transitions between multiple steady states. Varying the parameters in the network model to increase the height of the ridges relative to the valleys dramatically reduced the probability of transitions between the steady states (Figure 5), even though there was no appreciable change in the relative distance between the steady states on the *x-y *phase plane (Figure 4A-D, right panels).

The "third dimension" (elevation) of the landscape represented by the quasi-potential, although directly derived from the dynamic rate equations without any additional information, thus yields an interpretation of cellular stability not immediately apparent from two-dimensional phase portrait analysis. The analysis above supports the contention that the length of the "least action trajectory" along the contours of the epigenetic landscape is more important in predicting transitions between alternative cellular states than the simple "aerial distance" in state space [13]. It is also interesting to note that the contours of the quantitatively mapped epigenetic landscape act as a constraint on the extent of stochastic fluctuations in protein levels, with simulated cells "smeared out" on the surface of a shallower basin (Figure 4A, middle panel) compared to a tighter distribution of cells on a deeper valley (Figure 4D, middle panel).

These results suggest that calculating the relative heights of the ridges and valleys on the computed epigenetic landscape of a multi-gene system can help predict the probability of trans-differentiation from one cell lineage to another, or de-differentiation of a particular cell type to its progenitor state. Current efforts to reprogram cell fate with potential application in regenerative medicine suffer from a low rate of successful reprogramming [36] and a trial-and-error approach to choice of a reprogramming strategy [13]. Computing the epigenetic landscape for the critical gene interactions regulating the transition between two cellular states may indicate particular genetic manipulations that would lower the barriers separating the two states, thereby increasing the efficiency of the reprogramming process. It can also help characterize the relative ease or difficulty of alternative routes of cell fate transition [7,9,36]. For instance, comparison of the elevation of the barriers separating two terminally-differentiated cell lineages on the epigenetic landscape might suggest that de-differentiation of cells of one lineage to the common progenitor cell of the two lineages followed by redirection to the second lineage would lead to more efficient reprogramming than direct trans-differentiation (Figure S2, Additional File 1).

### A dynamic landscape

**Figure 6:**
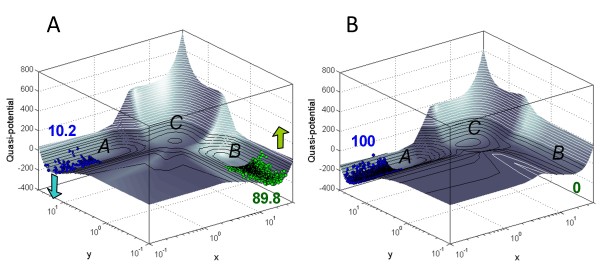
**The shape of the computed epigenetic landscape can be altered by modifying gene interaction parameters**. When basal expression *B*_*y *_of gene *y *in the tristable two-gene system (see **Methods**) is increased from *B*_*y *_= 0 **(A) **to *B*_*y *_= 4 **(B) **(dimensionless units), attractor *A *on the landscape is "lowered" relative to attractor *B*, causing cells to "roll into" the more stable state *A *from the destabilized state *B*. Numbers on the figure refer to the percentage of stochastically simulated cells in the respective attractors. All simulations were started from state *B *as the initial condition, and run to time *t *= 10,000. Hill coefficient *n*_*H *_= 10 in both figures.

The computed epigenetic landscape derived above should not be interpreted as a static surface [45]. Alterations in gene interactions in course of development or experimental manipulation will change the shape of the landscape, in turn altering the stability of individual steady states or creating novel steady states. For instance, increasing the basal expression of one gene in the tristable gene network sharply lowers the elevation of the corresponding attractor state relative to the other attractors (Figure 6A, B). As a result, cells located in the shallower attractor are destabilized and "roll into" the valley representing the deeper, more stable attractor state. This may explain the phenomenon of trans-differentiation of cells of one lineage into another by forced expression of a gene regulating the second lineage or by conditional deletion of a gene required for the first lineage [29,30].

Interestingly, this flexibility of the quasi-potential surface under gene manipulation gives a quantitative interpretation of the revised image of the epigenetic landscape proposed by Waddington (Figure S3, Additional File 1), which showed an array of pegs representing genes, holding up a sheet of fabric (the landscape) through a network of guy ropes (gene interactions) - meant to convey the idea that "the modelling of the epigenetic landscape ... is controlled by the pull of these numerous guy-ropes which are ultimately anchored to the genes [5]". Similar changes in the shape of the epigenetic landscape may also be brought about by external signals - for example endogenous cytokines or environmental chemicals - which by transiently altering the landscape could have an instructive effect on cell fate choice.

## Conclusions

In this work, we have defined a deterministic quasi-potential that is minimized along a temporal trajectory followed by a gene network, and used it to quantitatively derive the corresponding epigenetic landscape. A gene network not being a mechanical system, this quasi-potential should not be confused with a potential energy function. It is rather a Liapunov function of the dynamical system represented by the gene network, along which trajectories flow monotonically "downhill" towards the steady states of the network [41]. Other investigators have used a term analogous to the quasi-potential difference Δ *V*_*q *_in Eq. 4 to calculate the "energy landscape" for concentrations of one component in a gene network [46,47]. Here we have used the concept of alignment of multiple trajectories to interpolate the epigenetic landscape of a two-variable system.

This novel and simple process for deriving the surface of the landscape from a path-integral quasi-potential is not restricted to two-gene systems. While the landscape cannot be visually rendered for circuits with more than two genes, the rates of transition across the potential barriers between multiple steady states in the system can still be computed to predict optimum routes of cell fate reprogramming.

However, many binary branching points in development, particularly in blood cell lineage specification, are governed by mutual antagonism of only two transcription factors associated with alternative lineage choices [37]. Mapping the epigenetic landscape of pairs of such cross-inhibitory "master regulators" should therefore be of particular interest in understanding both normal development and induced cell fate reprogramming, and can be greatly aided by detailed quantitative characterization of the interactions between these regulators.

## Methods

### Bistable network model

To illustrate the derivation of the epigenetic landscape, we used a simplified mathematical model of a bistable network of two genes, *x *and *y*, that suppress each other to form a double-negative feedback loop. The dynamics of the model are described by the two rate equations:(6)(7)

where variables *x *and *y *represent the concentrations of the two gene products, and parameters *B*_*X *_and *B*_*Y *_denote the basal (constitutive) expression rates of genes *x *and *y*, respectively. The parameters *fold*_*YX *_and *fold*_*XY *_represent the rate constants, and *K*_*DYX *_and *K*_*DXY *_the effective affinity constants, for the suppressive effects of gene *y *on gene *x*, and of gene *x *on gene *y*, respectively. The mutual suppression of the two genes is quantified by the Hill-coefficient *n*_*H *_(the interaction is *ultrasensitive *for values of *n*_*H *_> 1). Parameters *deg*_*X *_and *deg*_*Y *_represent the first-order degradation rate constants for the two gene products *x *and *y*, respectively. For this simplified model we used dimensionless parameters with the following values: *fold*_*YX *_= *fold*_*XY *_= 2; *K*_*DYX *_= 0.7; *K*_*DXY *_= 0.5; *B*_*X *_= *B*_*Y *_= 0.2; *deg*_*X *_= *deg*_*Y *_= 1; *n*_*H *_= 4. These values were tuned to ensure bistable switching behavior in the model.

### Tristable network model

The tristable network model consisted of two genes, *x *and *y*, that in addition to mutual suppression, induce their own expression (positive autoregulation). The dynamics of this model are described by:(8)(9)

where the new parameters *fold*_*XX *_and *fold*_*YY *_represent the rate constants, and *K*_*DXX *_and *K*_*XYY *_the effective affinity constants, for the positive autoregulation of genes *x *and *y*, respectively. The default parameter values chosen to ensure three robust stable states in this model were as follows: *fold*_*XX *_= *fold*_*YY *_= *fold*_*YX *_= *fold*_*XY *_= 10; *K*_*DXX *_= *K*_*DYY *_= *K*_*DYX *_= *K*_*DXY *_= 4; *B*_*X *_= *B*_*Y *_= 0; *deg*_*X *_= *deg*_*Y *_= 1; *n*_*H *_= 4. This system has been modeled previously [42,48,49] in the context of mutual inhibition of the transcription factors PU.1 and GATA1 in common myeloid progenitor (CMP) cells, which gives rise to either bipotential granulocyte/macrophage progenitor (GMP) cells or megakaryocyte/erythroid progenitor (MEP) cells.

### Integration Algorithm

To evaluate the change in the quasi-potential along each trajectory in *x-y *phase space by numerical integration, the initial level of the quasi-potential at time *t *= 0 at the origin of the trajectory was arbitrarily set to zero (the same initial quasi-potential level was used for all trajectories so that the drop in the quasi-potential along each trajectory could be compared and used as a basis for alignment of multiple trajectories along a basin of attraction).

Thereafter, at each time step:

• The rates  and  were updated to the current value of *x *and *y *according to Eqs. 8 and 9.

• Expression levels *x *and *y *were updated as:(10)(11)

where for increments in time Δ*t *(fixed for a simulation to ensure convergence), the changes in *x *and *y *are given by:(12)(13)

• The quasi-potential *V*_*q *_was updated as:(14)

where:(15)

The above steps were repeated until the quasi-potential *V*_*q *_converged to a minimum (decided by a pre-set tolerance). Multiple trajectories thus obtained were aligned into basins of attraction according to the process described in the main text. The quasi-potential surface was then derived by linear interpolation among the aligned trajectories.

### Software platforms used

The deterministic models were implemented and simulated on the MATLAB^® ^(R2009a, The MathWorks, Inc., Natick, MA) platform, while the BioNetS program [50], based on the Gillespie algorithm [51,52], was used for stochastic simulations. All graphics were rendered on MATLAB^®^.

### Visualization of stochastic simulation results

The stochastically simulated "cells" (i.e. individual realizations of the stochastic network model) were overlaid on the quasi-potential surface at the *x *and *y *values predicted for each cell. Since stochastic simulations yield integral values, we added a small random "deviation" term [= (rand*0.5) where *rand *is a MATLAB^® ^function that draws pseudorandom values from the standard uniform distribution on the open interval (0,1)] to each simulated *x *and *y *value to visualize multiple cells situated at the same point in *x-y *phase space. The appropriate "elevation" for each cell on the quasi-potential surface was calculated by linear interpolation between the two points on the deterministic trajectories "closest to" the location of the cell in *x-y *phase space. Source code for the model in MATLAB^® ^format is appended in Additional File 2.

## Authors' contributions

SB designed the study, constructed the computational model and performed computer simulations, and wrote the paper. QZ participated in study design. QZ and MEA discussed the results and commented on the manuscript. All authors read and approved the final manuscript.

## Supplementary Material

Additional file 1 **Supplementary Figures**. This file includes additional figures to supplement the text. Click here for file

Additional file 2 **Supplementary Model Code**. This file lists the source code in MATLAB^® ^format for the computational algorithm used to derive the epigenetic landscape. Click here for file
