# Supplementary material for: A deterministic map of Waddington's epigenetic landscape for cell fate specification
Source: BMC Syst Biol. 2011 May 27;5:85. doi: 10.1186/1752-0509-5-85 (PMC3213676; doi:10.1186/1752-0509-5-85)
Supplement: Additional file 1 — Supplementary Figures. This file includes additional figures to supplement the text. [file 1752-0509-5-85-S1.DOC]

**Supplementary Figures**

**A deterministic map of Waddington’s epigenetic landscape for cell fate specification**

### Sudin Bhattacharya, Qiang Zhang and Melvin E. Andersen


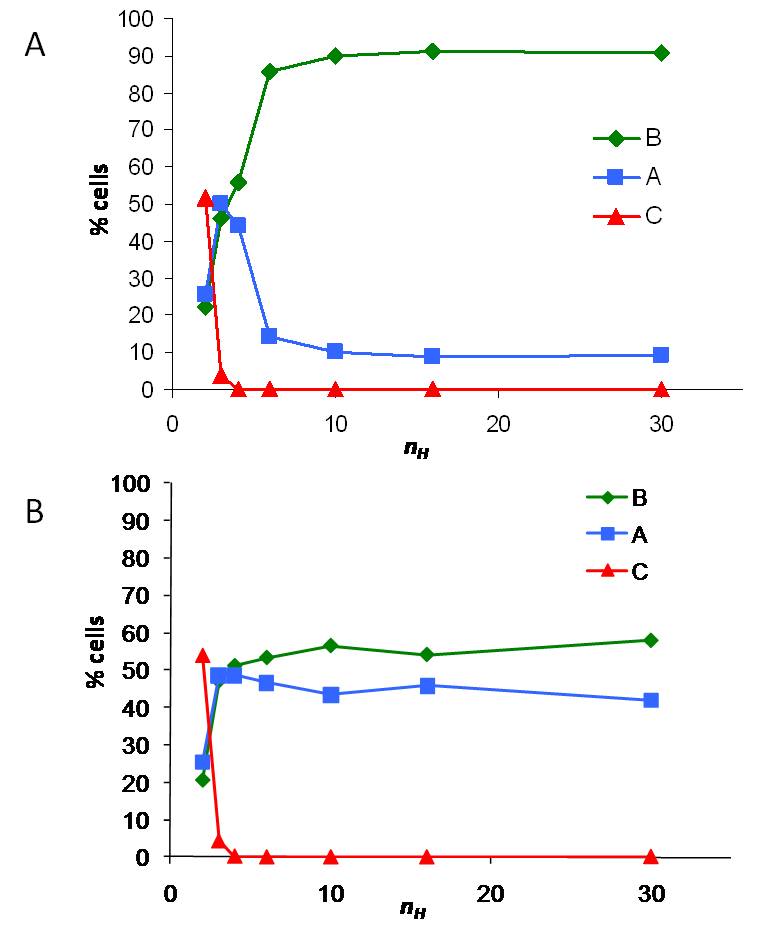


**Fig. S1**. **Percentage of stochastically simulated cells in the three attractors *A, B* and *C* for different values of *n_H_*, at time scales (A) *t* = 10,000; and (B) *t* = 100,000.** All simulations were started from state *B* as the initial condition.

At a shorter time scale *t* = 10,000 (panel **A**), greater barrier height with increasing *n_H_* reduced the likelihood of stochastic transitions from state *B* to state *C*, and thereafter to state *A* – thus keeping most cells in attractor *B*. However at the longer time scale *t* = 100,000 (panel **B**), the percentage of cells in each attractor correlated simply to the relative depth of the attractor (attractors *A* and *B* are deeper than attractor *C* for *n_H_* > 2: see Fig. S2).


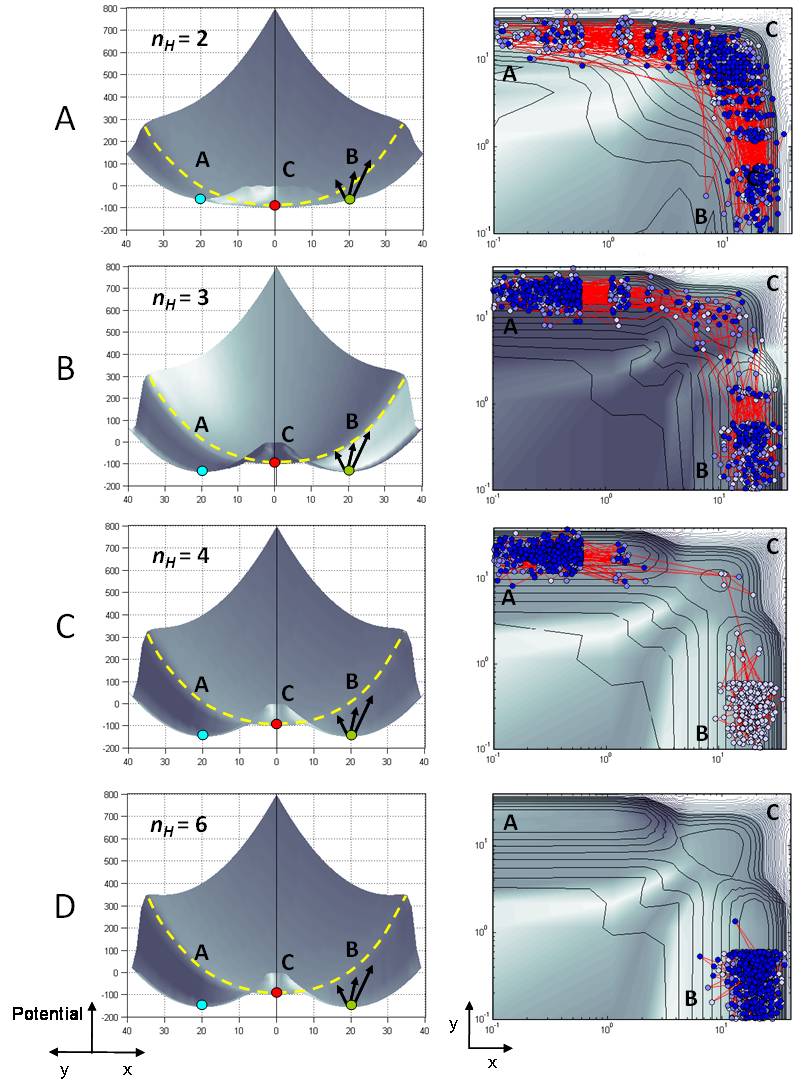


**Fig. S2**. **Stochastic transitions among multiple steady states of a tristable two-gene circuit are reduced by higher barriers on the computed epigenetic landscape. (A)** *n_H_* = 2; **(B)** *n_H_* = 3; **(C)** *n_H_* = 4; **(D)** *n_H_* = 6.

Panels on left show schematic relation between the location of stable steady states *A, B* and *C*, and the barriers separating these states (dashed yellow lines). States *A* and *B* correspond to differentiated cell fates, and state *C* to their common progenitor state [1]. Stochastic transitions (arrows, left panels) from state *B* to states *C* and *A* become progressively less likely with increasing barrier height and steepness. Panels on right track the trajectory of a single stochastically simulated cell on the computed epigenetic landscape. All simulations are started from state *B* as initial condition. Circles represent different time points on the trajectory, with earlier times shaded in light blue and later times in darker blue. Red lines denote the transitions between time points, thus tracking the evolution of a trajectory over time. Note that for *n_H_* = 2 (panels **A**), the intermediate state *C* is more stable than states *A* or *B*, since it is located at a lower elevation on the epigenetic landscape. The preferred route of transitions from state *B* to state *A* (right panel of **C**) is through the intermediate (progenitor) state *C*.


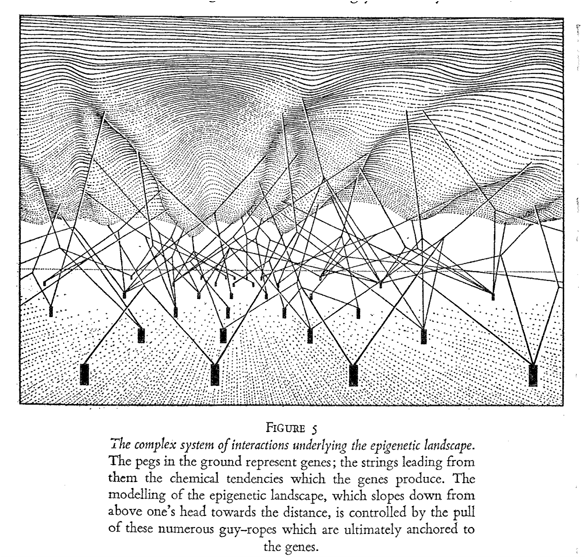


**Fig. S3**. **Waddington’s image of the view “under” the epigenetic landscape.** The “pull of these numerous guy-ropes … anchored to the genes” can be interpreted as the gene interaction network that controls “the modelling of the epigenetic landscape”. Figure reproduced from original text by Waddington [2].

**References**

1. Huang S, Guo YP, May G, Enver T: **Bifurcation dynamics in lineage-commitment in bipotent progenitor cells**. *Dev Biol* 2007, **305**(2):695-713.

2. Waddington CH: **The Strategy of the Genes**. London: George Allen & Unwin; 1957.
