# Supplementary material for: A deterministic map of Waddington's epigenetic landscape for cell fate specification
Source: BMC Syst Biol. 2011 May 27;5:85. doi: 10.1186/1752-0509-5-85 (PMC3213676; doi:10.1186/1752-0509-5-85)
Supplement: Additional file 2 — Supplementary Model Code. This file lists the source code in MATLAB® format for the computational algorithm used to derive the epigenetic landscape. [file 1752-0509-5-85-S2.DOC]

**Supplementary Model Code**

**A deterministic map of Waddington’s epigenetic landscape for cell fate specification**

### Sudin Bhattacharya, Qiang Zhang and Melvin E. Andersen

%%%%%%%%%%%%%%%%%%%%%%%%%%%%%%%%%%%%%%%%%%%%%%%%%%%%%%%%%%%%%%%%%%%%%%%%

%--- **MATLAB®** Code to plot "potential surface" for two-gene tristable switch system ---

%

% Deterministic rate equations

% dxdt = c1 + a1*(x^n)/(Kdxx^n + (x^n)) + (b1*(Kdyx^n))/(Kdyx^n + (y^n)) - (x*k1)

% dydt = c1 + a2*(y^n)/(Kdyy^n + (y^n)) + (b2*(Kdxy^n))/(Kdxy^n + (x^n)) - (y*k2)

%

% -- Working version --

%

% created by Sudin Bhattacharya 6/1/10

%%%%%%%%%%%%%%%%%%%%%%%%%%%%%%%%%%%%%%%%%%%%%%%%%%%%%%%%%%%%%%%%%%%%%%%%

clear all;

tic; %track run time

%Parameters

n = 4;

a1 = 10.0;

a2 = 10.0;

Kdxx = 4;

Kdyx = 4;

Kdyy = 4;

Kdxy = 4;

b1 = 10.0;

b2 = 10.0;

k1 = 1.0;

k2 = 1.0;

c1 = 0;

c2 = 0;

%%%%%%%%%%%%%%%%%%%%%%%%%%%%%%%%%%%%%%%%%%%%%%%%%%%%%%%%%%%%%%%%%%%%%%%%

% -- First, generate potential surface from deterministic rate equations –

% Define grid spacing for "starting points" for each "path" on the pot. surface

xyGridSpacing = 2;

x_lim = 40.0; % upper limit of x, y (zero to ...)

y_lim = 40.0;

% No. of time steps for integrating along each path (to ensure uniform arrays)

numTimeSteps = 1400; %Choose high-enough number for convergence with given dt

% Time step and tolerance to test for convergence

dt = 1.0e-2; % ** Check convergence for assigned "numTimeSteps" and tol

tol = 1.0e-4; % **

% Calculate total no. of paths for defined grid spacing

numPaths = 0;

for i = 0 : xyGridSpacing : x_lim

for j = 0 : xyGridSpacing : y_lim

numPaths = numPaths + 1;

end

end

% Initialize "path" variable matrices

x_path = zeros(numPaths,numTimeSteps); %x-coord. along path

y_path = zeros(numPaths,numTimeSteps); %y-coord. along path

pot_path = zeros(numPaths,numTimeSteps); %pot. along path

path_tag = ones(numPaths,1); %tag for given path (to denote basin of attraction)

% ** initialized to 1 for all paths **

% Initialize "Path counter" to 1

path_counter = 1;

% Initialize no. of attractors and separatrices (basin boundaries)

num_attractors = 0;

num_sepx = 0;

% Assign array to keep track of attractors and their coordinates; and pot.

attractors_num_X_Y = [];

attractors_pot = [];

% Assign array to keep track of no. of paths per attractor

numPaths_att = [];

% Assign array to keep track of separatrices

sepx_old_new_pathNum = [];

%%%%%%%%%%%%%%%%%%%%%%%%%%%%%%%%%%%%%%%%%%%%%%%%%%%%%%%%%%%%%%%%%%%%%%%%

% Loop over x-y grid

for i = 0 : xyGridSpacing : x_lim

for j = 0 : xyGridSpacing : y_lim

%*** Init conds for given (x,y) ***

%Initialize coords.

x0 = i;

y0 = j;

% ** Set initial value of "potential" to 0 **

p0 = 0; % (to facilitate comparison of "potential drop")

%Initialize "path" variables

x_p = x0;

y_p = y0;

%Initialize accumulators for "potential" along path

Pot = p0;

Pot_old = 1.0e7; %initialize to large number

%Initialize global arrays (time t = 0 counts as "time step #1")

x_path(path_counter, 1) = x_p;

y_path(path_counter, 1) = y_p;

pot_path(path_counter, 1) = Pot;

%Evaluate potential (Integrate) over trajectory from init cond to

stable steady state

for n_steps = 2:numTimeSteps

%record "old" values of variables

%x_old = x_p;

%y_old = y_p;

%v_old = v;

Pot_old = Pot;

%update dxdt, dydt

dxdt = c1 + a1*(x_p^n)/(Kdxx^n + (x_p^n)) + (b1*(Kdyx^n))/(Kdyx^n +

(y_p^n)) - (x_p*k1);

dydt = c2 + a2*(y_p^n)/(Kdyy^n + (y_p^n)) + (b2*(Kdxy^n))/(Kdxy^n +

(x_p^n)) - (y_p*k2);

%update x, y

dx = (c1 + a1*(x_p^n)/(Kdxx^n + (x_p^n)) + (b1*(Kdyx^n))/(Kdyx^n +

(y_p^n)) - (x_p*k1))*dt;

dy = (c2 + a2*(y_p^n)/(Kdyy^n + (y_p^n)) + (b2*(Kdxy^n))/(Kdxy^n +

(x_p^n)) - (y_p*k2))*dt;

x_p = x_p + dx;

y_p = y_p + dy;

x_path(path_counter, n_steps) = x_p;

y_path(path_counter, n_steps) = y_p;

%update "potential"

dPot = - (dxdt)*dx - (dydt)*dy; % signs ensure that "potential"

decreases as "velocity" increases

Pot = Pot_old + dPot;

pot_path(path_counter, n_steps) = Pot;

end % end integration over path

%check for convergence

if (abs(Pot - Pot_old) > tol)

fprintf(1,'Warning: not converged!\n');

end

% --- assign path tag (to track multiple basins of attraction) ---

if (path_counter == 1)

%record attractor of first path and its coords

num_attractors = num_attractors + 1;

current_att_num_X_Y = [num_attractors x_p y_p]; %create array

attractors_num_X_Y = [attractors_num_X_Y; current_att_num_X_Y];

% append array (vertically)

attractors_pot = [attractors_pot; Pot];

% append attractor potentials to array (vertically)

path_tag(path_counter) = num_attractors; %initialize path tag

numPaths_att = [numPaths_att; 1]; % append to array (vertically)

else % i.e. if path counter > 1

%set path tag to that of previous path (default)

path_tag(path_counter) = path_tag(path_counter - 1);

%record info of previous path

x0_lastPath = x_path((path_counter - 1), 1);

y0_lastPath = y_path((path_counter - 1), 1);

xp_lastPath = x_path((path_counter - 1), numTimeSteps);

yp_lastPath = y_path((path_counter - 1), numTimeSteps);

pot_p_lastPath = pot_path((path_counter - 1), numTimeSteps);

%calculate distance between "start points" of current and previous

paths

startPt_dist_sqr = ((x0 - x0_lastPath)^2 + (y0 - y0_lastPath)^2);

%calculate distance between "end points" of current and previous

paths

endPt_dist_sqr = ((x_p - xp_lastPath)^2 + (y_p - yp_lastPath)^2);

%check if the current path *ended* in a different point compared to

previous path

% (x-y grid spacing used as a "tolerance" for distance)

if ( endPt_dist_sqr > (2 * (xyGridSpacing^2)) )

% --- check if this "different" attractor has been identified

before

new_attr_found = 1;

for k = 1 : num_attractors

x_att = attractors_num_X_Y(k,2);

y_att = attractors_num_X_Y(k,3);

if ( (abs(x_p - x_att) < xyGridSpacing) &&

(abs(y_p - y_att) < xyGridSpacing) )

% this attractor has been identified before

new_attr_found = 0;

path_tag(path_counter) = k; % DOUBLE CHECK ***

numPaths_att(k) = numPaths_att(k) + 1;

break; %exit for-loop

end

end

if (new_attr_found == 1)

num_attractors = num_attractors + 1;

current_att_num_X_Y = [num_attractors x_p y_p];

%create array

attractors_num_X_Y = [attractors_num_X_Y;

current_att_num_X_Y];

% append array (vertically)

path_tag(path_counter) = num_attractors; % DOUBLE CHECK **

numPaths_att = [numPaths_att; 1];

% append to array (vertically)

%%%% check if start points of current and previous paths

are "adjacent"

% - if so, assign separatrix

if (startPt_dist_sqr < (2 * (xyGridSpacing^2)))

curr_sepx = [path_tag(path_counter - 1)

path_tag(path_counter) (path_counter - 1)];

%create array

sepx_old_new_pathNum = [sepx_old_new_pathNum;

curr_sepx];

% append array (vertically)

attractors_pot = [attractors_pot; Pot];

% append attractor potentials to array

(vertically)

num_sepx = num_sepx + 1;

% increment no. of separatrices

end

else

% --- check if the attractor of the *previous* path

% has been encountered in a separatrix before ---

% (note that current path tag has already been set

% above)

prev_attr_new = 1;

for k = 1 : num_sepx

attr1 = sepx_old_new_pathNum(k,1);

attr2 = sepx_old_new_pathNum(k,2);

if ( (path_tag(path_counter - 1) == attr1) || ...

(path_tag(path_counter - 1) == attr2) )

% this attractor has been identified before

prev_attr_new = 0;

break; %exit for-loop

end

end

if (prev_attr_new == 1)

%%%% check if start points of current and previous

paths are "adjacent"

% - if so, assign separatrix

if (startPt_dist_sqr < (2 * (xyGridSpacing^2)))

curr_sepx = [path_tag(path_counter - 1)

path_tag(path_counter) (path_counter - 1)];

%create array

sepx_old_new_pathNum = [sepx_old_new_pathNum;

curr_sepx];

% append array (vertically)

attractors_pot = [attractors_pot; pot_p_lastPath];

% append attractor potentials to array

(vertically)

num_sepx = num_sepx + 1;

% increment no. of separatrices

end

end

end

else %i.e. current path converged at same pt. as previous path

%%update path tag

%path_tag(path_counter) = path_tag(path_counter - 1);

%update no. of paths for current attractor

% (path tag already updated at start of path-counter loop)

tag = path_tag(path_counter);

numPaths_att(tag) = numPaths_att(tag) + 1;

end % end check for location of path end-pt.

end

% increment "path counter"

path_counter = path_counter + 1;

end

end

fprintf(1,' Ran path-loop okay!\n');

%%%%%%%%%%%%%%%%%%%%%%%%%%%%%%%%%%%%%%%%%%%%%%%%%%%%%%%%%%%%%%%%%%%%%%%%

% -- need 1-D "lists" (vectors) to plot all x,y, Pot values along paths --

list_size = numPaths*numTimeSteps;

x_p_list = zeros(list_size,1);

y_p_list = zeros(list_size,1);

pot_p_list = zeros(list_size,1);

n_list = 1;

%%%%%%%%%%%%%%%%%%%%%%%%%%%%%%%%%%%%%%%%%%%%%%%%%%%%%%%%%%%%%%%%%%%%%%%%

%"Align" potential values so all path-potentials end up at same global min.

for n_path = 1:numPaths

tag = path_tag(n_path);

del_pot = pot_path(n_path, numTimeSteps) - attractors_pot(tag);

%align pot. at each time step along path

for n_steps = 1:numTimeSteps

pot_old = pot_path(n_path, n_steps);

pot_path(n_path, n_steps) = pot_old - del_pot;

%add data point to list

x_p_list(n_list) = x_path(n_path, n_steps);

y_p_list(n_list) = y_path(n_path, n_steps);

pot_p_list(n_list) = pot_path(n_path, n_steps);

n_list = n_list + 1; % increment n_list

end

end

fprintf(1,' Ran path-alignment okay!\n');

fprintf(1,' ************************\n');

%%%%%%%%%%%%%%%%%%%%%%%%%%%%%%%%%%%%%%%%%%%%%%%%%%%%%%%%%%%%%%%%%%%%%%%

%%%%%%%%%%%%%%%%%%%%%%%%%%%%%%%%%%%%%%%%%%%%%%%%%%%%%%%%%%%%%%%%%%%%%%%

% Generate surface interpolation grid

% % To generate log-log surface

% x_p_list = x_p_list + 0.1;

% y_p_list = y_p_list + 0.1;

%--- Create X,Y grid to interpolate "potential surface" ---

grid_lines = 45; %No. of grid lines in x- and y- directions

xlin = linspace(min(x_p_list), max(x_p_list), grid_lines);

ylin = linspace(min(y_p_list), max(y_p_list), grid_lines);

[Xgrid,Ygrid] = meshgrid(xlin,ylin);

Zgrid = griddata(x_p_list, y_p_list, pot_p_list, Xgrid, Ygrid, 'linear');

fprintf(1,' Ran surface grid-interpolation okay!\n');

fprintf(1,' ************************************\n');

%%%%%%%%%%%%%%%%%%%%%%%%%%%%%%%%%%%%%%%%%%%%%%%%%%%%%%%%%%%%%%%%%%%%%%%%

%%%%%%%%%%%%%%%%%%%%%%%%%%%%%%%%%%%%%%%%%%%%%%%%%%%%%%%%%%%%%%%%%%%%%%%%

% ------ Plot "potential surface" ------------------

figure(1)

hold on;

surfl(Xgrid, Ygrid, Zgrid);

shading interp

colormap bone;

grid on;

box on;

xlabel('x', 'fontsize',12)

ylabel('y', 'fontsize',12)

zlabel('Potential', 'fontsize',12)

% -- Plot contour lines --

hold on;

contour3(Xgrid, Ygrid, Zgrid, 45);

%%%%%%%%%%%%%%%%%%%%%%%%%%%%%%%%%%%%%%%%%%%%%%%%%%%%%%%%%%%%%%%%%%%%%%%%

% -- Plot selected paths on pot. surface --

path_spacing = 4;

hold on;

for n_path = 1:numPaths

if ( ((mod(x_path(n_path, 1), path_spacing) == 0) && (mod(y_path(n_path, 1), path_spacing) == 0)) ...

|| ((mod(y_path(n_path, 1), path_spacing) == 0) && (mod(x_path(n_path, 1), path_spacing) == 0)) )

% % *** To generate log-log surface ***

% x_path(n_path, :) = x_path(n_path, :) + 0.1;

% y_path(n_path, :) = y_path(n_path, :) + 0.1;

% % ***

if (path_tag(n_path) == 1)

plot3(x_path(n_path, :), y_path(n_path, :), pot_path(n_path, :) ...

, '-r' , 'MarkerSize', 1) % plot paths

elseif (path_tag(n_path) == 2)

plot3(x_path(n_path, :), y_path(n_path, :), pot_path(n_path, :) ...

, '-b' , 'MarkerSize', 1) % plot paths

elseif (path_tag(n_path) == 3)

plot3(x_path(n_path, :), y_path(n_path, :), pot_path(n_path, :) ...

, '-g' , 'MarkerSize', 1) % plot paths

end

hold on;

end

end

%%%%%%%%%%%%%%%%%%%%%%%%%%%%%%%%%%%%%%%%%%%%%%%%%%%%%%%%%%%%%%%%%%%%%%%%

%%%%%%%%%%%%%%%%%%%%%%%%%%%%%%%%%%%%%%%%%%%%%%%%%%%%%%%%%%%%%%%%%%%%%%%%

%--- Run stochastic simulations ---

% Define # of cells

num_of_cells = 10000;

% Initialize arrays to store x-y end-data; and calculated "potential"

x_array = zeros(num_of_cells, 1);

y_array = zeros(num_of_cells, 1);

pot_array_stoch = zeros(num_of_cells, 1);

% counters for cells in each basin of attraction

num_cells_blue = 0;

num_cells_red = 0;

num_cells_green = 0;

% Initialize arrays to store path (list) indices

list_index1 = zeros(num_of_cells, 1);

list_index2 = zeros(num_of_cells, 1);

%--- begin loop over number of cells ---

for i = 1:num_of_cells

fprintf(1, 'cell #: %i\n', i);

load ('C:\Program Files\bionets 2.0\WorkingDirs\

TriStableSwitch_newParams\Input.mat');

%load Input.mat;

Seed = 10000*unifrnd(0,1);

BetweenSaves = 100;

%Save back only what belongs to the original Input.mat.

save Input.mat BetweenSaves Epsilon Implicit K MaximumTime Seed dt x -v4;

%run the simulation (each call to runme.exe invokes the BioNetS program)

% *** Need a copy of updated runme.exe in current directory ***

!runme.exe;

%load the simulation result

load ('C:\Program Files\bionets2.0\WorkingDirs\

TriStableSwitch_newParams\Output.mat');

% --- Read in Steady State vals from time series ---

% transform data to enable log plot and ...

% add random "background deviation" to distinguish overlapping data points

x_endVal = Series(2,end)+ (0.1 + rand*0.5);

y_endVal = Series(3,end)+ (0.1 + rand*0.5);

% Store x-y data

x_array(i) = x_endVal;

y_array(i) = y_endVal;

% record stoch. X-Y data

x_stoch = x_array(i);

y_stoch = y_array(i);

distSqr = 1.0e7; %initialize to high value

% run through path-(x,y) list to find the two points on the pot. surface

% "closest" to stoch X-Y point

for j = 1:list_size

% record path X-Y data

x_det = x_p_list(j);

y_det = y_p_list(j);

if ((x_stoch - x_det)^2 + (y_stoch - y_det)^2) < distSqr

list_index2(i) = list_index1(i);

distSqr = (x_stoch - x_det)^2 + (y_stoch - y_det)^2;

list_index1(i) = j;

end

end

% now interpolate between the two closest path points to evaluate

% "potential" level of the stochastic X-Y data point

% (assume linear variation of potential in this small interval)

x_path_1 = x_p_list(list_index1(i));

x_path_2 = x_p_list(list_index2(i));

y_path_1 = y_p_list(list_index1(i));

y_path_2 = y_p_list(list_index2(i));

pot_path_1 = pot_p_list(list_index1(i));

pot_path_2 = pot_p_list(list_index2(i));

%*********************

%Linear interpolation

%*********************

x1x2_tol = 1.0e-2;

y1y2_tol = 1.0e-2;

% Check for division by zero (or small term)

if ((x_path_2 - x_path_1) >= x1x2_tol)

p_stoch = ((x_stoch - x_path_1)/(x_path_2 - x_path_1)) * (pot_path_2 –

pot_path_1) + pot_path_1;

elseif ((y_path_2 - y_path_1) >= y1y2_tol)

p_stoch = ((y_stoch - y_path_1)/(y_path_2 - y_path_1)) * (pot_path_2 –

pot_path_1)+ pot_path_1;

else

p_stoch = pot_path_1;

end

pot_array_stoch(i) = p_stoch; % record interpolated potential

end

%%%%%%%%%%%%%%%%%%%%%%%%%%%%%%%%%%%%%%%%%%%%%%%%%%%%%%%%%%%%%%%%%%%%%%%%

%------- Plot stoch. data on pot. surface -----

fprintf(1,'Plotting stoch data...\n');

hold on;

%--- begin loop over number of cells ---

for i = 1:num_of_cells

% --- Plot data colored by basin of attraction

if ((x_array(i) < 5) && (y_array(i) > 5))

plot3(x_array(i), y_array(i), pot_array_stoch(i), 'ob', ...

'MarkerSize', 5, 'MarkerEdgeColor','k','MarkerFaceColor','b');

num_cells_blue = num_cells_blue + 1;

elseif ((x_array(i) > 5) && (y_array(i) < 5))

plot3(x_array(i), y_array(i), pot_array_stoch(i), 'og', ...

'MarkerSize', 5, 'MarkerEdgeColor','k','MarkerFaceColor','g');

num_cells_green = num_cells_green + 1;

else

plot3(x_array(i), y_array(i), pot_array_stoch(i), 'or', ...

'MarkerSize', 5, 'MarkerEdgeColor','k','MarkerFaceColor','r');

num_cells_red = num_cells_red + 1;

end

hold on;

end

% end of program

%

%%%%%%%%%%%%%%%%%%%%%%%%%%%%%%%%%%%%%%%%%%%%%%%%%%%%%%%%%%%%%%%%%%%%%%%%

%%%%%%%%%%%%%%%%%%%%%%%%%%%%%%%%%%%%%%%%%%%%%%%%%%%%%%%%%%%%%%%%%%%%%%%%
